# Industry Perceptions of Thoroughbred Racehorse Emotions and Quality of Life: Implications for the Development of an Equine Quality of Life Assessment Tool

**DOI:** 10.3390/ani16111601

**Published:** 2026-05-25

**Authors:** Jane M. Williams, Cathrynne Henshall, Natalie Waran, Hayley Randle

**Affiliations:** 1 Equine Department, Hartpury University, Hartpury, Gloucestershire GL19 3BE, UK; 2 School of Agricultural, Environmental and Veterinary Sciences, Charles Sturt University, Wagga Wagga, NSW 2678, Australia; chenshall@csu.edu.au (C.H.); hrandle@csu.edu.au (H.R.); 3 NavigateWelfare, 743 Puketitiri Road, Napier 4182, New Zealand; nat@navigatewelfare.com

**Keywords:** Thoroughbred, horse welfare, quality of life, emotions, positive affective state, racing industry

## Abstract

Understanding how horses experience their lives is essential for improving their welfare, particularly in high-performance industries such as horseracing. Quality of Life (QoL) frameworks highlight the balance between positive and negative experiences over time, yet there is limited evidence on how racing professionals assess horse emotions and QoL, especially positive emotional states. This study reports findings from an international online survey of 230 racing industry participants. It explored confidence in assessing racehorse emotions, the perceived importance of emotional state for performance and welfare, views on racehorse QoL, and changes in understanding over time. Most respondents (92.5%) reported high confidence in judging racehorse emotions, and 94.8% of these considered emotional state important or very important for performance. The majority (87.7%) believed racehorses experience a good life at least sometimes, although this varied depending on management practices, individual horse characteristics, and human factors. Turnout, social contact, health management, staff experience, and regulatory environments were identified as key influences on QoL. Recognition of the importance of emotional state and QoL within the industry is increasing, alongside a shift towards more individualized, welfare-focused approaches. These findings support developing practical, industry-informed emotional state measures within equine QoL tools for assessing horses’ experiences and inform management decisions.

## Simple Summary

Understanding how horses feel in their daily lives is important for improving their welfare, especially in industries like horse racing. This study looked at how people (stable staff and trainers) working with racehorses assess horses’ emotions and overall quality of life. An online survey of 230 racing industry participants from around the world asked about their confidence in judging racehorse emotions, how important they think these emotions were for performance and welfare, and whether they believed racehorses experienced a good life. Most respondents said they felt confident in recognizing horse emotions, and nearly all believed these emotions were important for performance. Many participants thought racehorses experienced a good life at least some of the time, but their answers showed that this could vary depending on how horses were managed, their individual needs, and the people caring for them. Key factors influencing a racehorse’s quality of life included time spent outdoors, opportunities to interact with other horses, good health care, skilled staff, and appropriate rules and regulations. Overall, the findings suggest growing awareness within the horseracing industry of the importance of equine emotions and welfare and support the development of practical tools to help people better understand and improve horses’ lives.

## 1. Introduction

Horse welfare fundamentally concerns how horses experience their lives, including their ability to cope with challenges and benefit from opportunities. Contemporary animal welfare science increasingly recognises that welfare cannot be adequately assessed solely through the absence of negative states such as pain, fear, or distress, but must also consider the presence of positive experiences and emotions [1,2,3]. This reflects a significant shift in animal welfare science, moving beyond the traditional Five Freedoms framework towards the more comprehensive Five Domains model across the last decade [2]. The Five Domains model provides a contemporary approach to welfare assessment by integrating a broader range of internal and external factors, including physical state, environment, and human interactions. Importantly, it extends beyond the prevention of suffering to include the promotion of positive experiences, with explicit consideration of the animal’s affective state and emotional responses [3]. The balance of positive and negative affective experiences over time underpins an animal’s Quality of Life (QoL), encompassing physical, psychological, and environmental dimensions of welfare [4]. Within this context, the Five Domains model offers an appropriate framework for assessing racehorses’ quality of life, as it captures both the mitigation of negative states and the promotion of positive affective experiences [3].

In horses, and particularly within the Thoroughbred racing industry, welfare assessments have traditionally focused on physical health, performance metrics, and overt indicators of stress or discomfort [5,6,7]. Validated behavioural indicators exist for negative affective states such as pain and discomfort [8,9,10]. However, despite recent studies [11,12,13], there remains a paucity of validated, practicable indicators of positive emotional states in horses [14]. This is problematic, as the absence of negative indicators does not necessarily equate to good welfare or a good quality of life [15].

Advances in affective neuroscience and animal welfare science demonstrate that emotional states influence health, learning, motivation, and performance across species [16,17,18]. In equine contexts, affective or emotional state may also have implications for rider and handler safety, trainability, and athletic performance [19,20]. Consequently, there is growing interest in identifying reliable indicators of both negative and positive affect that can be used by industry practitioners to support welfare-positive management decisions.

Whether racehorses experience a good life before, during, and after their racing careers is a focus for both the public and industry stakeholders [5,21,22,23,24,25]. Recent work undertaking welfare assessment of racehorses in Great Britain has demonstrated the value of combining animal-based indicators with management data to provide an evidence-based understanding of welfare in training environments [5]. While the results found most horses generally exhibited good physical health, notable variation existed in management practices such as social contact, turnout, and feeding, all of which are closely linked to emotional state. Furthermore, behavioural evaluation undertaken by industry assessors (mostly non-veterinarians and non-scientists with experience of working with racehorses) reported most horses responded positively to human interaction, but a minority exhibited behaviours associated with compromised welfare, indicating potential negative emotional states [5]. These findings reinforce that racehorse welfare extends beyond physical health to include emotional wellbeing. Therefore, generating data that can evaluate how horses feel during different experiences across racing activities is critical to implement responsible practices and ethical standards that can optimise Thoroughbred health and welfare.

Horseracing workforce demographics reveal persistent global challenges in recruitment and retention. In Britain, the sector indirectly supports over 85,000 people, with approximately 7961 registered racing staff caring for more than 20,000 horses [26]. In 2024, the Racing Foundation’s Racing Industry Recruitment, Skills and Retention report estimated a racing staff and jockey population of 1591, with 6% of permanent posts requiring annual recruitment for growth, 45% of trainers reporting hard-to-fill vacancies, and staff turnover at 23% [27]. Migrant workers comprise an estimated 30–40% of the British workforce, concentrated in larger flat yards [28]. These patterns are mirrored internationally; in the United States, migrant workers predominantly from South America fulfil essential stable staff roles, while Australia and New Zealand similarly depend on migrant labour for skilled stable and track work personnel. Across all jurisdictions, skill shortages, declining retention rates, and concerns for workforce physical and mental health are acknowledged as sector-wide challenges, underscoring the international horseracing industry’s reliance on mobile and migrant workforces to sustain operations [29,30]. Given this demographic diversity, understanding how staff from varied cultural and linguistic backgrounds interpret and manage racehorse welfare is essential; consequently, simple, translatable tools that support consistent assessment of racehorse QoL and that can be practically embedded into training and daily practice across all communities and at all levels of the sector are vital to ensuring equitable welfare standards across the global industry.

The development of an Equine Quality of Life (EQoL) assessment tool suitable for use in the racing industry requires an understanding of how emotions and QoL are currently perceived and assessed by those working most closely with racehorses. Industry engagement is also critical for the successful implementation of welfare assessment tools, with tools that align with practitioner experience and priorities more likely to be adopted and used consistently. The overarching aim of the broader research programme that this study is a part of is to characterise the expression of emotion, particularly positive affective states, in Thoroughbred racehorses to contribute to the development of an EQoL assessment tool. This study specifically aimed to (1) identify how racing industry practitioners currently assess positive and negative emotional states in Thoroughbred horses; (2) to explore stakeholder perception of racehorse quality of life; and (3) examine whether stakeholder views on equine emotions have changed over time.

## 2. Materials and Methods

This study was approved by the Hartpury University Ethics Committee (ETHICS2022-27). All participants were provided with written information outlining the aims and rationale of the study and gave informed consent prior to participation.

### 2.1. Survey Design

An anonymous online survey was developed using Qualtrics XM™ (Seattle, WA, USA). The survey was designed to gather industry stakeholder perceptions of emotions, feelings, and their contribution to racehorse quality of life, drawing on respondents’ prior experience and views rather than aligning to a predefined definition. This approach enabled sector-specific colloquialisms and inherent cultural perspectives to emerge, capturing the internal industry understanding of racehorse quality of life. It included 21 questions which were a mixture of multiple-choice items (*n* = 6), Likert scale questions (*n* = 8), and open-ended free-text questions (*n* = 7) designed to capture both quantitative trends and qualitative insights (Supplementary File S1). Likert scale items assessed levels of agreement (rated as essential, important but not essential or not essential) or perceived importance of proposed positive and negative emotions within racehorses (rated as very important (1), moderately important (2), important (3), sometimes important, (4) not important (5)) with an option for respondents to indicate uncertainty where appropriate. The survey was open for 4 months (1 November 2023 to 28 February 2024).

The survey was pilot tested by four independent experienced equine researchers to ensure clarity, relevance, and face validity. Minor edits were made prior to deployment. The final survey link was distributed via racing industry stakeholder organisations and relevant social media platforms across global racing jurisdictions (Great Britain and Ireland, Australia and New Zealand, the United States and Canada, Middle East, Hong Kong, and Japan, such as Horseracing Social and Horseracing Jobs UK) using convenience and snowball sampling. The survey was distributed in English and eligibility criteria required respondents to be aged 18 years or older and to have worked with racehorses (within a training or track environment) for a minimum of 12 months.

The survey explored the following broad domains:

(1) Demographic information: including role within the racing industry, geographical location, and years of experience.
(2) Assessment of emotions: confidence in assessing equine emotions and perceived importance of emotional state for performance.
(3) Racehorse emotions: perceptions of which emotions racehorses experience and how these relate to mood and performance.
(4) Quality of life: views on racehorse quality of life, including whether racehorses are perceived to experience a good life and what factors influence this.
(5) Respondent knowledge and understanding of equine emotions: perceived changes in respondent understanding of racehorse emotions over the past decade and which sources of information respondents choose to access to inform their knowledge and understanding of emotions.

### 2.2. Data Analysis

Data were exported from Qualtrics to Microsoft Excel™ version 2010 (Redmond, WA, USA). Data for two descriptors: “Playful” and “Distressed” were removed prior to analysis due to issues with the survey software. Frequency analysis identified the demographic characteristics of respondents. Likert scale responses for respondent ratings were converted into numerical values in Microsoft Excel (version 25) to enable the calculation of the median rating response for each emotion as rated by respondent roles: trainer, assistant trainer, head lad/girl, stable/yard staff or strapper and ‘other’ roles, and by respondent experience within the horse racing industry: 0–2 yrs, 3–5 yrs, 6–10 yrs, 11–15 yrs and >20 yrs, prior to analysis. Data met non-parametric assumptions, therefore a series of Kruskal–Wallis analyses identified if differences occurred in how respondents rated specific emotions as indicators of positive or negative emotional states in racehorses by role and experience in racing. Where significant differences were found, Mann–Whitney U post hoc tests with appropriate adjustment for multiple testing identified how ratings differed between respondent roles and years of experience. Median rankings for individual emotions were examined to identify the direction of differences between role and experience in racing. Where median values were the same, mean rank differences obtained from post hoc tests differentiated between roles. Data were processed using SPSS version 29 and significance was set at *p* < 0.05.

Content validity ratios (CVRs) were calculated to determine if respondents agreed on which emotions indicated positive and negative emotional states in racehorses using established thresholds to confirm agreement exceeded chance [21,31,32]. Consensus was defined *a priori* as agreement by ≥70% of respondents (critical value: 0.7, where positive values exceeding the critical value confirming consensus that a factor is essential and negative values exceeding the critical value confirming consensus that a factor is not essential) [33,34]. The CVR was calculated using:

CVR = (ne − N/2)/((N/2)),

where “CVR” is content validity ratio, “ne” is the number of respondents who rated an emotion as essential, and “N” is the number of respondents who answered the questions [32,34].

To determine how much respondents agreed on which emotions represented overall positive and negative emotional states in racehorses, the average level of agreement for each category was calculated afterwards using the content validity index (CVI) [35,36]. To obtain the CVI for positive and negative emotional states, the number of respondents judging the item as essential (CVR) was divided by the total number of emotions being assessed [37]:

*CVI* = *∑C*V*R*/n

where “CVR” is content validity ratio value for individual emotions in a defined area, and “n” is the number of emotions assessed for positive and negative affective state respectively (with the two omitted descriptors: “Playful” and “Distressed” removed).

Open questions within the survey were evaluated through inductive conventional content analysis within the broader framework of McClelland’s Acquired-Needs Theory using tags (‘open-coding’) via a grounded theory approach to identify emergent themes arising from the data [38,39]. JW and HR undertook this analysis as experienced equine welfare scientists, with a commitment to evidence-based practice. Their positionality reflects a belief that welfare and performance are implicitly linked, recognising that optimising the horse’s physical and psychological wellbeing is fundamental to sustainable athletic achievement. While supportive of the use of horses in sport, they prioritise the provision of an overall good life, aligning with contemporary frameworks emphasising positive experiences and thriving. A pragmatic epistemological stance underpins the analysis, valuing practical, context-sensitive knowledge that bridges scientific inquiry with real-world applicability, while maintaining reflexive awareness of how professional experience may shape data interpretation.

## 3. Results

### 3.1. Participant Demographics

**Figure 1:**
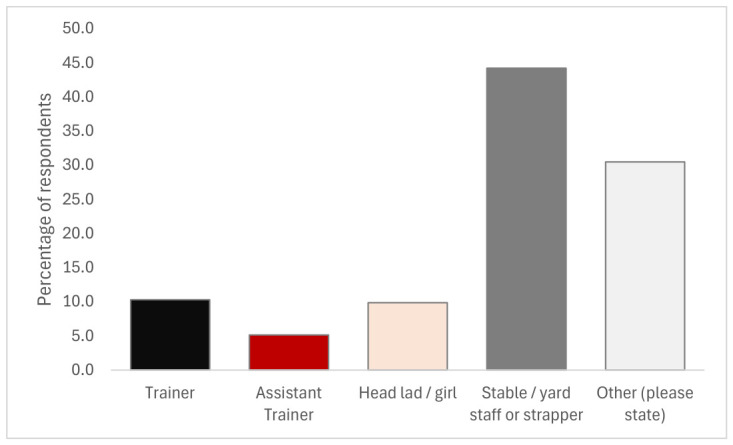
Distribution of self-selected respondent roles within the racing industry (*n* = 233); the top 3 ‘other’ roles were (1) stud staff/breeder, (2) work rider/jockey, and (3) racing administrator/management. Stable staff/groom/strapper: a term that refers to a male or female worker of any age in the racing industry, responsible for the daily care of the horses within the yard. They are traditionally referred to as stable lads, or stable lasses for female employees, and colloquialism of strapper is applied to these roles. Head person/lad/girl/lass/groom: they may also be referred to as yard manager, this role involves ensuring the yard operates smoothly daily. They may be responsible for horses’ feeding regimes, general health, and welfare checks or assisting the veterinarian with ongoing treatments.

**Figure 2:**
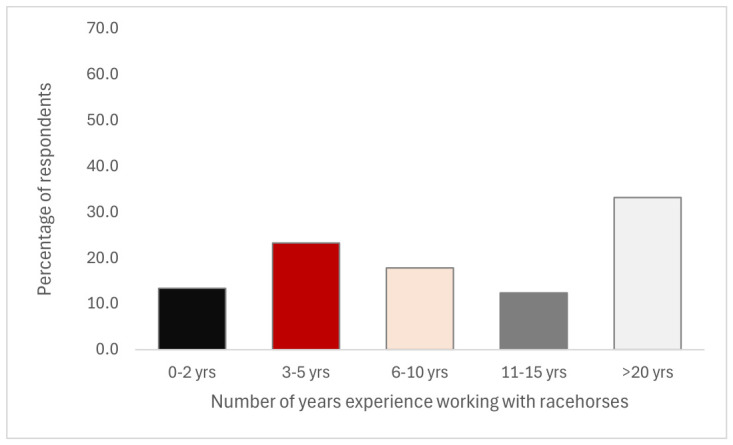
Number of years of experience working with racehorses, self-reported by respondents (*n* = 202).

The 233 respondents who completed the survey represented various roles in the racing industry (Figure 1). Most were based in Great Britain and Ireland (48.1%; *n* = 112) or Australia and New Zealand (42.1%; *n* = 98), with the remainder resident in other global racing jurisdictions. Over 85% had worked with racehorses for more than three years, suggesting that the sample largely comprised experienced practitioners with protracted exposure to racehorse management and training environments (Figure 2).

### 3.2. Confidence-Assessing Racehorse Emotions

Most respondents self-reported high confidence in their ability to judge how a racehorse is feeling. Over 90% (92.6%; *n* = 216) indicated that they felt confident they knew enough about equine emotions to assess emotional state, while only a small minority expressed uncertainty (5.1%; *n* = 11) or lack of confidence (2.6%; *n* = 6). Perceived importance of emotional state underpinning equine performance was also high. Most respondents rated how a horse is feeling as important or very important for racing performance (very important: 84.7% (*n* = 194); moderately important 3.5% (*n* = 8); important 11.4% (*n* = 26)), with only a negligible proportion (0.4%; *n* = 1) considering emotional state to be unimportant.

### 3.3. Perceptions of Impacts of Emotions on Racehorse Performance

**Figure 3:**
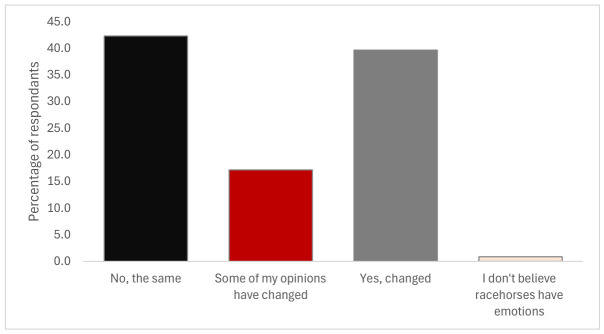
Respondent self-reported view if their opinion on what emotions racehorses feel and/or experience has changed in the last 10 years (*n* = 227).

Respondents were divided regarding whether their own self-reported opinions about racehorse emotions had changed over the past 10 years, with roughly similar proportions indicating change had occurred versus remaining stable (Figure 3). Qualitative analysis of free-text responses revealed two dominant themes emerged, which could underpin changes in respondents’ perspectives: (1) increased personal experience and knowledge, and (2) a broader cultural shift within the racing industry (Table 1). Many respondents described how accumulated experience improved their (self-perceived) ability to interpret equine behaviour, recognize pain-related or stress-related changes, and appreciate individual differences between horses. Others emphasized they perceived an industry-wide shift towards greater awareness of welfare, sentience, and the emotional lives of horses.

Respondents noted a shift (in their own opinion) across the industry from viewing racehorses being regarded only as athletes towards acknowledging them as individuals with emotional needs. They also noted that they believed the racing industry had developed an increased awareness of horse welfare, emotions, and quality of life. They felt that they believed increasingly over the past 10 years, horses were being treated more as individuals with individual needs. They also believed that the link between pain and behaviour and performance was better understood and that there is an increased perception in the racing industry that horses have feelings, are sentient, and should not be treated as machines.

### 3.4. Participant Views on Equine Quality of Life

Most respondents believed that racehorses experience a ‘good life’: always 11.3% (24/212), sometimes 76.4% (162/212); whilst 10.8% (23/212) felt racehorses did not have a good life and 1.4% (3/212) were not sure. However, qualitative responses highlighted substantial variability in understanding of the term ‘Quality of Life’, with many linking it heavily to management practices, the competence and attitudes of staff, and the suitability of individual horses for racing (Table 2).

Whilst respondents agreed that emotions labelled ‘happiness’, ‘excitement’, ‘distress’, and ‘fear’ were indicative of how a racehorse was feeling but views on other emotions varied (Table 3).

Equine behaviours such as eating well, coming when called, and being active and engaged in their work as well as positive human interactions were agreed by respondents to indicate a ‘good mood’, while a horse which was dull in its demeanour or that was not eating well was agreed to reflect a bad mood (Table 4).

### 3.5. Participant Self-Reported Knowledge and Understanding of Equine Emotions

The importance given to knowledge of different emotions and interactions indicative of whether a horse is in a good or bad mood did not differ between respondent roles for most factors evaluated. The only exception was the extent to which poor appetite indicated that a racehorse was in a negative emotional state, which showed a significant difference (*p* = 0.001). Post hoc analysis found that trainers (median ± IQR: 1 ± 2) rated this indicator as less important than assistant trainers (median ± IQR: 1 ± 0; *p* = 0.02), stable/yard staff, and strappers (median ± IQR: 1 ± 0, *p* = 0.0001) and those employed in other roles in racing (median ± IQR: 1 ± 0, *p* = 0.002).

Overall, respondents consistently rated the extent to which emotions and interactions reflected a racehorse’s emotional state similarly, regardless of experience. Only a few differences emerged: ratings for insecurity (*p* = 0.015) and fear (*p* = 0.007) varied initially, but these differences were not significant in post hoc analyses. Perceptions of wariness did differ by experience (*p* = 0.02), with those having 6–10 years’ experience (median ± IQR: 1 ± 0) in the industry viewing it as more indicative of a horse’s emotional state than those with 3–5 years’ experience (median ± IQR: 1 ± 2). Importantly, there were no differences across roles or experience in how respondents rated the influence of emotions on racehorse performance.

**Figure 4:**
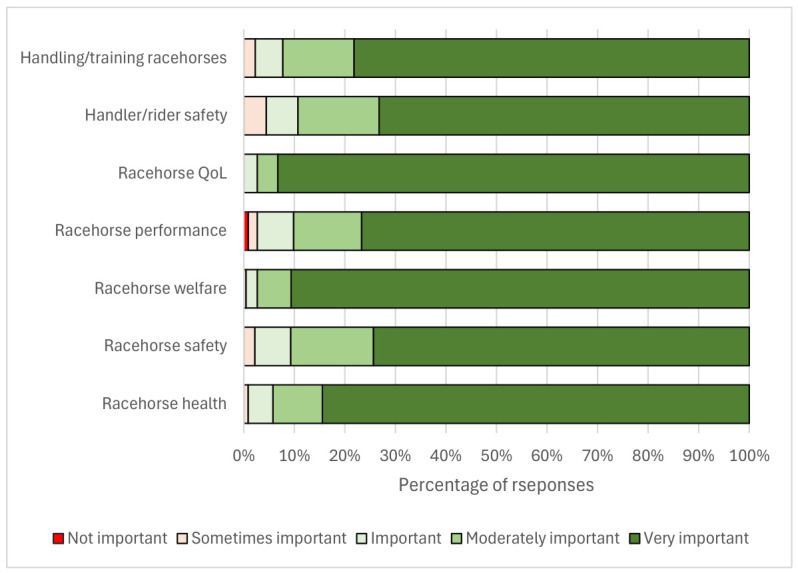
Respondent self-rating (*n* = 226) the importance that racehorses do experience a positive affective state within evaluation of racehorse and racing staff health, safety, and performance. QoL: Quality of Life.

**Figure 5:**
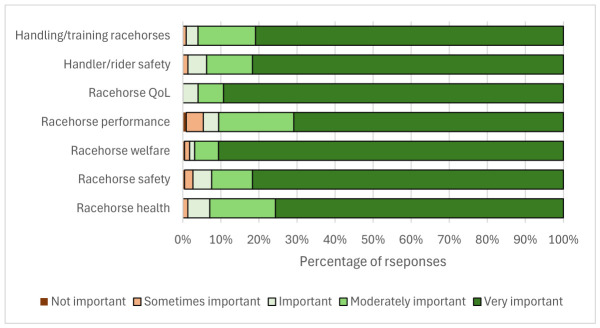
Respondent self-rating (*n* = 223) of importance that racehorses do not experience negative affective state within evaluation of racehorse and racing staff health, safety, and performance.

Most respondents consistently rated the need for racehorses to experience positive emotional states as important for their health and performance, and for horse and human safety (Figure 4 and Figure 5).

## 4. Discussion

This study provides insight into how Thoroughbred racing industry professionals perceive and assess racehorse emotions and equine quality of life based on their lived experience. The findings demonstrate that respondents self-report a high degree of confidence in their own ability to assess equine emotional states and a strong belief that affective state is integral to horse performance, safety, and welfare. These perceptions align with contemporary animal welfare science, which highlights the functional importance of affective states [7,40]. However, it should be noted that the accuracy of their evaluation has not been tested. In determining the relative importance of specific behaviours to identify how a horse is feeling, the CVR results suggest that participants rely on characteristics of the horse’s interactions with humans as well as performance linked attributes of horse behaviour such as being engaged in ridden work or appetence to assess emotional state. Less emphasis was placed on behaviours unrelated to these factors such as being restless in the stable, despite the latter behaviour likely being an important indicator of emotional state and overall quality of life [41].

Respondent role and the proximity to daily racehorse care and management could also influence how individuals rated the importance of factors. The horseracing workforce spans a wide range of roles and working arrangements, and in practice individuals may undertake multiple duties within the same yard (for example, supervisory staff may also ride out and/or provide direct horse care [29,30]). In this study limited respondent characteristic data focused on experience in racing and job role/title were collected rather than evaluating specific role responsibilities. As a result, it cannot be determined whether differences in respondent roles across yards or wider demographic variation (for example, gender, ethnicity, or cultural background) was represented in the respondent cohort, nor examine whether such factors shaped responses. Future work should aim to develop a clearer demographic profile of the racing workforce including ethnicity and gender, as well as systematically recording what individuals’ roles undertake for example, time in direct contact with horses, routine task allocation, and multi-role working, so that the potential influence of staff role, background, working context, and cultural influences on practice can be tested rather than hypothesized.

The evident widespread belief that racehorses experience a good life at least some of the time indicates a generally positive self-assessment of industry welfare standards. However, the qualitative responses revealed a more nuanced appreciation that QoL experienced by racehorses is highly variable and contingent on management quality, human attitudes, and individual horse needs. Research examining Thoroughbred racehorse management has consistently identified that horses in training are commonly housed in individual stables with limited opportunities for turnout and social interaction [42,43]. Geographic differences occur across global racing jurisdictions with equine husbandry in New Zealand reported to be more pasture-based [44] and increased urbanization in Australia appears to be influencing racehorse husbandry potentially limiting turnout and socialization opportunities compared to more traditional systems [45]. Differences in management practices experienced by respondents—specifically those in Great Britain and Ireland and Australia and New Zealand—could influence the results obtained. The variability observed also underscores the limitations of relying solely on structural or resource-based welfare indicators and supports the need for animal-based measures of emotional state to be integrated within QoL assessments [15]. Importantly, respondents described they perceived a shift in industry culture has taken place over the past decade towards greater recognition of equine sentience and emotional experience. This mirrors wider societal and scientific trends that prioritize positive welfare and quality of life as the expected standard for animal care, rather than simply meeting minimum requirements [3,46].

However, deficits in understanding what constitutes a “good life” for racehorses remain, reflected in the lack of agreement or consensus across many areas, an issue also identified in the wider sport horse community [6]. This may be further constrained by a tendency to rely primarily on health-based indicators, rather than incorporating broader measures of welfare including emotional state. Similar themes have been found in livestock farming, where a systematic review identified that farmers rank satisfying the biological function of an animal as the primary indicator of good welfare followed by expression of positive emotional states and expression of natural behaviours [47]. In an equestrian world where the horse–human relationship is becoming increasingly recognized, not least because it shapes outcomes for the horse, it is not surprising that this study provided evidence that industry participants recognize that human interactions can directly affect the emotional state of horses and their welfare.

Nearly all respondents recognized that positive welfare and a good quality of life in racehorses underpins optimal performance, whilst simultaneously acknowledging that tensions often exist within yards where management practices can limit factors such as turnout, social contact, or individualized regimes that are recognized to promote positive emotional states [48,49]. A small minority stated negative affective states did not impact racehorse performance (0.9%), safety (0.4%), and welfare (0.4%), suggesting some stakeholders do not fully recognize the importance of equine emotional wellbeing and QoL as central components of horse-centred care, and the core role this has within racing’s social licence to operate, and the long-term ethical sustainability of the racing industry. The role of stable staff and trainers in the management of the racehorses’ quality of life was consistently voiced, with respondents noting that a horse’s quality of life is highly dependent on the people responsible for horses’ daily care and the experience and empathy respondents reflected they possess. Prior work has shown that human–animal interactions in farming [50,51] and zoos [52,53] can influence the quality of care, emotional states, and welfare status of both the humans and animals involved. Respondents emphasized the importance of recognizing when a horse is no longer enjoying life as a racehorse. Indicators discussed included changes in performance, reduced enthusiasm during work, altered behaviour, and decreased interaction with humans, which respondents interpreted as potential signs of a negative affective state or reduced quality of life. While some of these indicators may appear performance-related, respondents generally framed them within a broader understanding of the horse’s emotional wellbeing and overall welfare, rather than solely in relation to racing output. This interpretation is supported by the finding that positive affective states were rated as important for welfare more consistently than for performance outcomes. Recognition of these behavioural and emotional changes may therefore help inform decisions regarding management, retraining, or retirement [21], and may reflect an emerging shift toward more horse-centred approaches within the racing industry.

### Limitations

This study reports self-reported survey data and as in all such studies, the data provided by respondents cannot be independently validated. Consequently, while respondents were generally confident in their ability to accurately identify emotional states in horses, findings from a range of studies have reported that even experienced horse industry participants may fail to accurately identify emotional state in horses [54,55,56]. The correct identification of positive emotions in practice may be no better than chance. Consequently, further research is required to validate the accuracy of emotional state detection by Thoroughbred industry.

It should be noted that the decision to prioritize ecological validity in the survey design was made deliberately to reflect the natural and colloquial ways in which racing industry stakeholders discuss racehorse emotions, behaviours, and welfare in practice, and to promote accessibility across a diverse respondent population, allowing the authentic cultural vocabulary and lived experience of the racing sector to emerge. However, this approach has introduced limitations to the study design. The absence of operational definitions or behavioural anchors for the affective states and the concept of “Quality of Life” presented to respondents could result in respondents interpreting terms according to their own professional experience, cultural understanding, or industry-specific vocabulary. The results could therefore capture semantic agreement and shared industry language as much as the recognition of clearly differentiated emotional states. Additionally, the mixed grammatical structure applied, which intentionally combined nouns and adjectives, may have increased cognitive load and affected response consistency. The simplification of complex qualitative descriptions into broader “good” and “bad” mood categories may not fully capture the distinction between transient affective states and longer-term welfare indicators such as stereotypic behaviours or chronic stress-related conditions. Additionally, interpretation and weighting of certain indicators, including behaviours such as “eating well”, may have been influenced by respondents’ professional roles and differing levels of day-to-day proximity to horses. This could potentially contribute to variation in how behavioural and welfare-related cues were perceived across stakeholder groups although the majority of respondents (>80%) were involved in ‘hands-on’ roles in the racing industry.

Given that the subjective experience of horses is critical to their welfare state [3], it is imperative that industry participants can accurately identify the emotional state of horses in their care. The present findings can help identify areas that practitioners already consider meaningful, and areas where validation that what practitioners describe aligns to agreed scientific definitions or interpretation, which can be used to guide future research priorities. Several role- and proximity-related mechanisms may help interpret these findings; for example, differences in horse contact and manual tasks. Prior work [29,30] has shown that stable staff roles tend to involve greater direct horse contact or more manual tasks than supervisory roles. However, as exposure or proximity to racehorses was not explicitly measured in the current study, inferences made between these and interpretation of racehorse wellbeing should be treated as hypothetical and future studies should aim to address this gap. Further research is also required to evaluate the influence of human-related factors, such as personal values and ethics, industry culture, proximity to the horse, and emotional attachment, on how racing industry staff identify and interpret racehorse emotional state. This will underpin the development of industry-focused resources, such as EQoL assessment tools, to inform decision-making to safeguard and enhance racehorse welfare.

The study may have been subject to social desirability and self-selection biases, as respondents who chose to engage with the survey may have held stronger interests or more favourable attitudes toward racehorse welfare and quality of life, or provided answers they perceived as socially or professionally acceptable rather than fully reflecting their personal views or practices. The high level of agreement observed across several measures relating to racehorse emotions and welfare may in part represent this bias, reducing the discriminatory variance and limiting the sensitivity of some analyses to detect more subtle differences in respondent perspectives. However, the consistently high agreement itself may reflect a genuine shared recognition of the importance of racehorse emotional wellbeing among industry stakeholders, particularly when considered alongside the nuanced and often critical perspectives expressed within the qualitative responses. It should also be noted that although consensus was assessed using established and validated quantitative approaches (e.g., CVR thresholds), ambiguity in industry terminology may mean that agreement does not necessarily indicate uniform interpretation across all respondents. The findings therefore represent a baseline indication of shared perspectives, rather than complete conceptual alignment. Future research should adopt more iterative or mixed-method approaches (e.g., Delphi methods or expanded qualitative exploration) to examine how these terms are interpreted in practice and how industry perceptions align with scientific assessment of racehorse welfare and affective state.

Cultural and value-based influences have been identified within the horseracing sector, including strong norms around prioritizing horse welfare [29]. While these factors provide a useful lens through which to interpret the present findings, they were not directly measured within this study. Future research should seek to explicitly measure these constructs to establish their role in shaping injury attitudes and responses within the racing workforce. The survey was also conducted exclusively in English, which may have influenced accessibility and engagement among migrant workers or participants for whom English is not a first language. This was not specifically measured within the present study and therefore the extent of any impact remains unknown. The recent British Horseracing Authority 2026 Ethnic and Cultural Diversity Independent Report [28], which specifically targeted ethnically and culturally diverse racing staff, did not identify language as a significant barrier to participation, suggesting that any effect on engagement in the current study may have been limited.

## 5. Conclusions

Racing industry professionals report high confidence in assessing racehorse emotions and believe horses’ emotional state impacts their performance and welfare. While most believe that racehorses can experience a good quality of life, they recognize that this is highly dependent on management practices, human factors, and individual horse needs. These findings demonstrate the relevance of developing industry-informed equine QoL assessment tools, with most respondents recognizing the importance of assessing racehorse emotional wellbeing and affective state to support horse welfare. However, the variation in how respondents interpreted and prioritized positive and negative affective states, while indicating a broad desire among stakeholders to support a positive quality of life for racehorses, demonstrate a need for greater clarity and consensus regarding the behavioural and emotional indicators that may reliably reflect how a horse is feeling. Consequently, further work is needed to collaboratively develop, test, and embed equine QoL assessment tools, including meaningful involvement of racing industry professionals, to support industry engagement and more accurate appraisal of equine affective states. Future work should focus on validating practical, reliable behavioural and physiological indicators that can quantify how a horse is feeling and evaluate their application within real-world racing environments.

## Figures and Tables

**Table 1:** Emergent themes derived from respondents who indicated their opinion on what emotions racehorses feel and/or experience has changed in the last 10 years. Inductive conventional content analysis using a grounded theory approach identified emergent themes arising from the data. Bold text denotes a higher order theme.

**Increased personal** **experience and knowledge**	Increased personal recognition of racehorse emotional experiences.
	Developing knowledge through experiential learning. Links between ‘happiness’ and performance. Shifts in perceptions and ability to identify positive and negative aspects of the sector.
	Increased recognition of racehorse emotional experiences.
**Cultural shift within the** **racing industry**	Horse as an individual. Recognition of [horse experiencing] fear, pain, confusion.
	Recognition that horses can form negative emotional associations. Increased welfare concerns related to traditional practice. Feeding, management, training.

**Table 2:** Emergent themes derived from respondent views on factors that influence racehorse quality of life through inductive conventional content analysis using a grounded theory approach to identify emergent themes arising from the data. Bold text denotes a higher order theme.

**Life is good vs. poor**	Depends on individual approach: “when done well = good life”.
	Traditional vs. progressive approach.
	Management factors: turnout, social contact, health.
	Individual and holistic approach vs. one size fits all.
**Influences on a good life**	Environment: country and facilities.
	Regulation (of yards).
	People: experience.
	People: horse’s life dependent on team—yard/trainer/staff.
**Horse**	Good welfare underpins good performance.
	Health.
	Suitability (if the horse does not enjoy activities undertaken or are not a good fit—retire/rehome).

**Table 3:** Respondent ratings of which emotions are important to evaluate how racehorses are feeling. Green shading indicates consensus (positive CVR values > 0.7 indicate agreement that respondents rated an area as essential and negative CVR values > −0.7 indicate agreement that respondents rated an area as not essential), yellow shading indicates agreement above CVI, and red shading indicates below CVI and a lack of agreement.

**Emotion** **CVI = 0.4**	**Happiness** **(*n* = 231)**	**Excitement** **(*n* = 229)**	**Enthusiasm** **(*n* = 225)**	**Calm** **(*n* = 230)**	**Affectionate** **(*n* = 228)**
Essential (%)	84.4	67.5	72.7	81.4	31.2
Important but not essential (%)	14.3	29.4	24.7	17.3	46.8
Not important (%)	1.3	2.2	0.0	0.9	20.8
CVR	0.7	0.7	0.5	0.6	−0.4
**Emotion**	**Sociable** **(*n* = 229)**	**Secure** **(*n* = 229)**	**Angry** **(*n* = 229)**	**Distressed** **(*n* = 230)**	**Sad** **(*n* = 229)**
Essential (%)	49.8	78.8	81.0	91.3	76.2
Important but not essential (%)	40.3	17.7	10.4	2.6	16.0
Not important (%)	9.1	2.6	7.8	5.6	6.5
CVR	0.0	0.6	0.6	0.8	0.5
**Emotion**	**Bored** **(*n* = 229)**	**Fearful** **(*n* = 229)**	**Frustrated** **(*n* = 229)**	**Lonely** **(*n* = 229)**	**Insecure** **(*n* = 229)**
Essential (%)	64.9	86.1	71.4	70.6	70.6
Important but not essential (%)	27.3	6.5	19.9	21.2	19.9
Not important (%)	6.9	6.5	7.8	7.4	8.7
CVR	0.3	0.7	0.4	0.4	0.4
**Emotion**	**Curiousness** **(*n* = 229)**	**Wariness** **(*n* = 229)**	**Contentment** **(*n* = 225)**	**Other emotions self-reported by respondents they use to assess racehorse mood**	
Essential (%)	36.8	56.7	73.6	Worry Nervousness Trusting Tension Confusion Stress/anxiety Separation anxiety Relaxed Pain Confidence	
Important but not essential (%)	54.1	37.2	22.9		
Not important (%)	6.5	3.5	0.9		
CVR	−0.2	0.2	0.5		

CVR: content validity ratio; CVI: content validity index.

**Table 4:** Respondent ratings for how important common horse behaviours and horse–human interactions are to evaluate if a racehorse is experiencing a good or bad mood. Green shading indicates consensus (positive CVR values > 0.7 indicate agreement that respondents rated an area as essential and negative CVR values > −0.7 indicate agreement that respondents rated an area as not essential), yellow shading indicates agreement above CVI and red shading indicates below CVI and a lack of agreement.

**Good Mood (CVI: 0.03)**					
**Behaviour/Interaction**	Horse approaches handlers with ears pricked	Horse whinnies/nickers when handler approaches	Horse extends its upper lip and wriggles it lip/s during grooming	Horse mutually grooms another horse	Horse comes when called
Essential (%)	54.9	29.8	26.8	43.6	16.2
Important but not essential (%)	42.9	51.8	50.9	43.6	49.8
Not important (%)	2.2	18.4	22.4	12.9	34.1
CVR	0.1	−0.4	−0.5	−0.1	−0.7
**Behaviour/Interaction**	Horse is enthusiastic in their work	Horse is active and engaged	Horse is eating well	Horse actively seeks human contact	Horse lies down in stable
Essential (%)	75.0	84.6	99.6	21.2	65.5
Important but not essential (%)	24.1	15.0	1.8	61.1	28.8
Not important (%)	0.9	0.4	0.0	19.0	5.8
CVR	0.5	0.7	1.0	−0.6	0.3
**Bad Mood (CVI: 0.5)**					
**Behaviour/interaction**	Horse actively avoids human contact	Horse bites/nips handler	Horse turns back on handler when they enter the stable or runs away when called	Horse has ears pinned back	Horse threatens to bite/kick when being handled or tacked up
Essential (%)	70.3	62.6	64.5	76.0	79.4
Important but not essential (%)	22.3	31.3	31.1	19.6	17.1
Not important (%)	7.4	6.2	4.4	4.4	3.5
CVR	0.4	0.3	0.3	0.5	0.6
**Behaviour/Interaction**	Horse is restless in the stable	Horse is active in the stable during feeding or rest periods	Horse is working below par	Horse is not eating well	Horse is dull
Essential (%)	67.5	46.7	80.6	93.4	86.3
Important but not essential (%)	28.5	43.6	15.9	5.7	11.9
Not important (%)	3.9	9.8	3.5	0.9	1.8
CVR	0.4	−0.1	0.6	0.9	0.7
Other indicators proposed by respondents that can indicate a racehorse is in a good/bad mood: Activity when in stable, when ridden, and during turnout. Head and neck position. Tail swishing. Head tossing. Stereotypical behaviours.					

CVR: content validity ratio; CVI: content validity index.

## Data Availability

The data presented in this study are available on request from the corresponding author due to ethical constraints.

## Supplementary Materials

The following supporting information can be downloaded at: https://www.mdpi.com/article/10.3390/ani16111601/s1, Supplementary File S1: Survey copy.

## Abbreviations

The following abbreviations are used in this manuscript:

n	Number
%	Percentage
CVR	Content validity ratio
CVI	Content validity index
EQOL	Equine Quality of Life
IQR	Inter quartile range
QOL	Quality of Life
TB	Thoroughbred
