# Supplementary material for: Industry Perceptions of Thoroughbred Racehorse Emotions and Quality of Life: Implications for the Development of an Equine Quality of Life Assessment Tool
Source: Animals (Basel). 2026 May 25;16(11):1601. doi: 10.3390/ani16111601 (PMC13255690; doi:10.3390/ani16111601)
Supplement: Supplementary file 1 [file animals-16-01601-s001.zip › animals-4287068-supplementary.pdf]

Brighter Side of Life Survey

Start of Block: Default Question Block

INTRODUCTION Thank you for choosing to complete our survey. This project aims to understand what existing measures are used in the racing industry to determine racehorse emotions (also known as positive / negative affective states). The survey should not take more than 5-10 minutes to complete. Please note that all information which is collected about you during the course of the research will be kept securely. The survey is anonymous and collects no identifiable personal data about you, so that you cannot be recognised from it. You will have the option to provide contact details to participate in future stages of the project; email addresses will be retained by the research team and will not be shared with any third parties. It is our intention to present the results within a final summary statement which will be presented at a relevant conference, published in an appropriate journal, and disseminated to a wider audience via articles in the equestrian lay press. This survey is part of a Hartpury University, Charles Sturt University, and EIT research project (ethics number: ETHICS2022-27). The research team comprises: Dr Jane Williams, Prof. Hayley Randle, Dr Cath Henshall and Prof. Natalie Waran. The project is being funded by the Hong Kong Jockey Club. If you have any questions or comments regarding this survey please use the box provided at the end of the survey or email Dr Jane Williams at jane.williams@hartpury.ac.uk Thank you again for supporting our work.

Consent It is up to you to decide whether or not to take part in this research. By filling out this questionnaire you are indicating your voluntary informed consent for your results to be included in data analysis. You are free to withdraw without giving a reason up the point of pressing submit at the end of the survey. I acknowledge that my participation in the study is voluntary, I am over 18 years of age, and that I provide consent for the data provided to be used for research purposes.

○ Yes

Q1 Please select the option which best represents your role or employment within the racing industry:

- ◯ Trainer (1)
- ◯ Assistant Trainer (2)
- ◯ Head lad / girl (3)
- ◯ Stable / yard staff or strapper (4)
- ◯ Other (please state) (5) ____

Q2 How long have you worked in / been involved with the racing industry?

- ◯ 0-2 years (1)
- ◯ 3-5 years (2)
- ◯ 6-10 years (3)
- ◯ 11-15 years (4)
- ◯ 16-20 years (5)
- ◯ more than 20 years (6)

Q3 Please select the racing jurisdiction where you work the most:

- ○ Great Britain including Ireland (1)
- ○ Europe (2)
- ○ USA and Canada (3)
- ○ Australia and New Zealand (4)
- ○ UAE (5)
- ○ Asia: China, Hong Kong and Japan (6)
- ○ Other (please state) (7)

We want your opinion on which areas should be used to assess how racehorses are feeling. In the following questions we have highlighted some initial areas which could be used and we would like to know whether or not you believe they should be applied. You will also be asked if there are any additional areas you would use to determine how a racehorse is feeling and we will ask you to briefly describe these. There are no right or wrong answers, our aim is to find out what you think, so please include anything you believe is relevant.

Q4 How critical (essential) is it to be able to recognise the following emotions in racehorses, to be able to assess how they are feeling?

	Essential (1)	Important but not essential (2)	Not essential (3)
Happiness (1)	○	○	○
Excitement (2)	○	○	○
Enthusiasm (3)	○	○	○
Calm (4)	○	○	○
Affectionate (5)	○	○	○
Playful (6)	○	○	○
Sociable (7)	○	○	○
Secure (8)	○	○	○
Angry (9)	○	○	○
Distressed (10)	○	○	○
Sad (11)	○	○	○
Bored (12)	○	○	○
Fearful (13)	○	○	○
Frustrated (14)	○	○	○
Lonely (15)	○	○	○
Insecure (16)	○	○	○

Alertness (17)	○	○	○
Curiousness (18)	○	○	○
Wariness (19)	○	○	○
Contentment (20)	○	○	○

Q5 Are there any other emotions that you feel are important to be able to recognise / assess when working with racehorses?

____

Q6 How essential do you feel the following areas are to be able to recognise that a racehorse is in a good mood?

	Essential (1)	Important but not essential (2)	Not essential (3)
Horse approaches handlers with ears pricked (1)	○	○	○
Horse whinnies / nickers when handler approaches (2)	○	○	○
Horse extends its upper lip and wriggles it lip/s during grooming (3)	○	○	○
Horse mutually grooms another horse (4)	○	○	○
Horse comes when called (5)	○	○	○
Horse lies down in stable (6)	○	○	○
Horse is enthusiastic in their work (7)	○	○	○
Horse is active and engaged (8)	○	○	○
Horse is eating well (9)	○	○	○
Horse actively seeks human contact (10)	○	○	○

Q7 How essential do you feel the following areas are to be able to recognise that a racehorse is in a bad mood?

	Essential (1)	Important but not essential (2)	Not essential (3)
Horse actively avoids human contact (1)	○	○	○
Horse bites / nips handler (2)	○	○	○
Horse turns back on handler when they enter the stable or runs away when called (3)	○	○	○
Horse has ears pinned back (4)	○	○	○
Horse threatens to bite / kick when being handled or tacked up (5)	○	○	○
Horse is restless in the stable (6)	○	○	○
Horse is active in the stable during feeding or rest periods (7)	○	○	○
Horse is working below par (8)	○	○	○
Horse is not eating well (9)	○	○	○
Horse is dull (10)	○	○	○

Q8 Do you use any other indicators in your daily work with racehorses to assess how they are feeling? Please list and describe these below.

____

Q9 Are you confident you know enough about equine emotions to be able to judge how a racehorse is feeling?

- ◯ Yes (1)
- ◯ No (2)
- ◯ I am not sure (3)

Q10 How important do you think it is to know how a horse is feeling, in relation to its performance as a racehorse?

- ◯ Very important (1)
- ◯ Important (3)
- ◯ Moderately important (2)
- ◯ Sometimes important (4)
- ◯ Not important (5)
- ◯ I am not sure (6)

Page Break

Q11 In your opinion, how important is the absence of negative emotions, e.g. fear, to each of the following areas:

	Very important (1)	Moderately important (2)	Important (3)	Sometimes important (4)	Not important (5)	I am not sure (6)
Racehorse health (1)	o	o	o	o	o	o
Racehorse safety (2)	o	o	o	o	o	o
Racehorse welfare (3)	o	o	o	o	o	o
Racehorse performance (4)	o	o	o	o	o	o
Racehorse quality of life (5)	o	o	o	o	o	o
Handler/ rider safety (6)	o	o	o	o	o	o
Handling and training racehorses (7)	o	o	o	o	o	o

Q12 In your opinion, how important is the presence of positive emotions, e.g. happiness, to each of the following areas:

	Very important (1)	Moderately important (2)	Important (3)	Sometimes important (4)	Not important (5)	I am not sure (6)
Racehorse health (1)	○	○	○	○	○	○
Racehorse safety (2)	○	○	○	○	○	○
Racehorse welfare (3)	○	○	○	○	○	○
Racehorse performance (4)	○	○	○	○	○	○
Racehorse quality of life (5)	○	○	○	○	○	○
Handler/ rider safety (6)	○	○	○	○	○	○
Handling and training racehorses (7)	○	○	○	○	○	○

Q13 Have you changed your opinions on what emotions racehorses feel / experience in the last 10 years?

- ○ Yes, changed (1)
- ○ Some of my opinions have changed (please explain how below) (4)
- ○ No, the same (2)
- ○ I don't believe racehorses have emotions (3)

Display this question:

If Have you changed your opinions on what emotions racehorses feel / experience in the last 10 years? = Yes, changed

Q13a Please can you describe how your opinions have changed:

____

Display this question:

If Have you changed your opinions on what emotions racehorses feel / experience in the last 10 years? = Some of my opinions have changed (please explain how below)

Q13a Please can you describe why and how some of your opinions have changed:

____

Q14 Please indicate how likely you would be to use the following potential sources of information relating to racehorse emotions if you wanted to find out more?

	Very likely (1)	Likely (2)	Sometimes Likely (3)	Unlikely (4)	I am not sure (5)
Books (1)	o	o	o	o	o
Word of mouth (2)	o	o	o	o	o
Social media (3)	o	o	o	o	o
Lay press e.g. magazines (4)	o	o	o	o	o
Veterinary professional (5)	o	o	o	o	o
Website (6)	o	o	o	o	o
Internet e.g., google search (7)	o	o	o	o	o
Experienced colleague (8)	o	o	o	o	o
Webinar/podcast (9)	o	o	o	o	o
Conference/ industry event attendance (10)	o	o	o	o	o
Behaviourist (11)	o	o	o	o	o
Equestrian professional (12)	o	o	o	o	o

Q15 Do you believe that racehorses have a good life?

- Yes, always (1)
- Yes, sometimes (2)
- No (3)
- I am not sure (4)

Q16 Please can you explain your answer:

____

Thank you for completing the survey. If you have any further comments or feedback on the survey, or any other comments regarding racehorse feelings and how these should be assessed, please use the space below to make these.

____

If you would like to take part in future research in this area, please include a contact email address below:

____

End of Block: Default Question Block
